# Inhibition of β2-Microglobulin/Hemochromatosis Enhances Radiation Sensitivity by Induction of Iron Overload in Prostate Cancer Cells

**DOI:** 10.1371/journal.pone.0068366

**Published:** 2013-07-10

**Authors:** Sajni Josson, Yasuhiro Matsuoka, Murali Gururajan, Takeo Nomura, Wen-Chin Huang, Xiaojian Yang, Jin-tai Lin, Roger Bridgman, Chia-Yi Chu, Peter A. Johnstone, Majd Zayzafoon, Peizhen Hu, Haiyen Zhau, Dror Berel, Andre Rogatko, Leland W. K. Chung

**Affiliations:** 1 Uro-Oncology Research Program, Department of Medicine, Samuel Oschin Comprehensive Cancer Institute, Cedars-Sinai Medical Center, Los Angeles, California, United States of America; 2 Molecular Urology and Therapeutics, Emory University School of Medicine, Atlanta, Georgia United States of America; 3 Hybridoma Facility, Auburn University, Auburn, Alabama, United States of America; 4 Radiation Oncology, Indiana University School of Medicine, Bloomington, Indiana, United States of America; 5 Department of Pathology, University of Alabama at Birmingham, Birmingham, Alabama, United States of America; 6 Biostatistics and Bioinformatics, Samuel Oschin Comprehensive Cancer Institute, Cedars-Sinai Medical Center, Los Angeles, California, United States of America; The University of Texas M.D Anderson Cancer Center, United States of America

## Abstract

**Background:**

Bone metastasis is the most lethal form of several cancers. The β2-microglobulin (β2-M)/hemochromatosis (HFE) complex plays an important role in cancer development and bone metastasis. We demonstrated previously that overexpression of β2-M in prostate, breast, lung and renal cancer leads to increased bone metastasis in mouse models. Therefore, we hypothesized that β2-M is a rational target to treat prostate cancer bone metastasis.

**Results:**

In this study, we demonstrate the role of β2-M and its binding partner, HFE, in modulating radiation sensitivity and chemo-sensitivity of prostate cancer. By genetic deletion of β2-M or HFE or using an anti-β2-M antibody (Ab), we demonstrate that prostate cancer cells are sensitive to radiation *in vitro* and *in vivo*. Inhibition of β2-M or HFE sensitized prostate cancer cells to radiation by increasing iron and reactive oxygen species and decreasing DNA repair and stress response proteins. Using xenograft mouse model, we demonstrate that anti-β2-M Ab sensitizes prostate cancer cells to radiation treatment. Additionally, anti-β2-M Ab was able to prevent tumor growth in an immunocompetent spontaneous prostate cancer mouse model. Since bone metastasis is lethal, we used a bone xenograft model to test the ability of anti-β2-M Ab and radiation to block tumor growth in the bone. Combination treatment significantly prevented tumor growth in the bone xenograft model by inhibiting β2-M and inducing iron overload. In addition to radiation sensitive effects, inhibition of β2-M sensitized prostate cancer cells to chemotherapeutic agents.

**Conclusion:**

Since prostate cancer bone metastatic patients have high β2-M in the tumor tissue and in the secreted form, targeting β2-M with anti-β2-M Ab is a promising therapeutic agent. Additionally, inhibition of β2-M sensitizes cancer cells to clinically used therapies such as radiation by inducing iron overload and decreasing DNA repair enzymes.

## Introduction

Prostate cancer bone metastasis is lethal. More than 70% of prostate cancer patients have bone metastasis at autopsy [1]. The median 5 year survival rate is only 31% for metastatic patients. Prostate cancer patients with bone metastasis have been shown to have high expression of β2-Microglobulin (β2-M) in the cancer cells [2]. β2-M is a cell membrane protein which complexes to the MHC class 1 family. β2-M is elevated in several aggressive solid and liquid tumors. It is a pleotropic factor which mediates multiple processes such as cancer development [3], cancer metastasis [4], and osteomimicry [2]. Previous studies demonstrate that targeting β2-M with anti-β2-M antibody (Ab) is a promising therapeutic strategy in prostate, renal and liquid tumors [5]–[7]. Previous studies demonstrate that β2-M interacts with hemochromatosis protein (HFE), which is a non-classical MHC class 1 member [8]. β2-M/HFE complex interacts with transferrin receptor (TFRC1), and lowers the affinity of transferrin binding to TFRC1 [9]. Thus, β2-M/HFE prevents excessive iron uptake. Mice lacking β2-M or HFE develop iron overload later in life and iron-related diseases [10], [11]. In this study we demonstrate that inhibition of β2-M using an antibody or genetic deletion of β2-M or HFE in cancer cells causes iron overload and sensitizes prostate cancer cells to radiation *in vitro* and *in vivo* and chemotherapeutic agents *in vitro*.

## Materials and Methods

### Bioethics Statement

All animal experiments were approved by the IACUC of the Emory University and the Cedars-Sinai Medical Center and done in accordance with institutional guidelines.

### Cell Culture

ARCaP_M_, ARCaP_E_ [12], C4-2, and C4-2B [13] prostate cancer cells were derived in our laboratory as described previously, and p69 (non-tumorigenic cells), LNCaP, PC-3, DU145, TRAMP C1 and TRAMP C2 prostate cancer cells were purchased from ATCC. Cells were cultured in T-medium (GibcoBRL, Grand Island, NY) supplemented with 5% heat inactivated fetal bovine serum (FBS) (Bio-Whittaker, Walkersville, MD), 50 IU/ml penicillin and 50 µg/ml streptomycin (GibcoBRL) and maintained in 5% CO_2_ at 37°C. All cells were tested for mycoplasma every six months and were negative (Mycoplasma detection kit, R&D systems).

### Cell Viability Assays

Clongenic assay was performed as previously mentioned [14]. Cell viability was determined with a CellTiter 96 Aqueous One Solution Cell Proliferation Assay (MTS assay) (Promega, Madison, WI).

### Radiation Studies

External beam radiation treatment was delivered on a 600 Varian linear accelerator with a 6 MV photon beam for *in vitro* and *in vivo* (subcutaneous and intra-tibial) experiments.

### Immunoblot Analysis

Western analysis was performed as previously described [2]. The membranes were incubated with mouse monoclonal antibody against β2-M, HFE, HSP27, HSP70 (Santa Cruz Biotechnology), NUDT1 and MPG (a gift from Dr. Yoke Wah Kow), EF-1α (Upstate), and β-actin (Sigma) respectively, at 4°C overnight.

### Anti-β2-M Ab Studies

**Figure 1:**
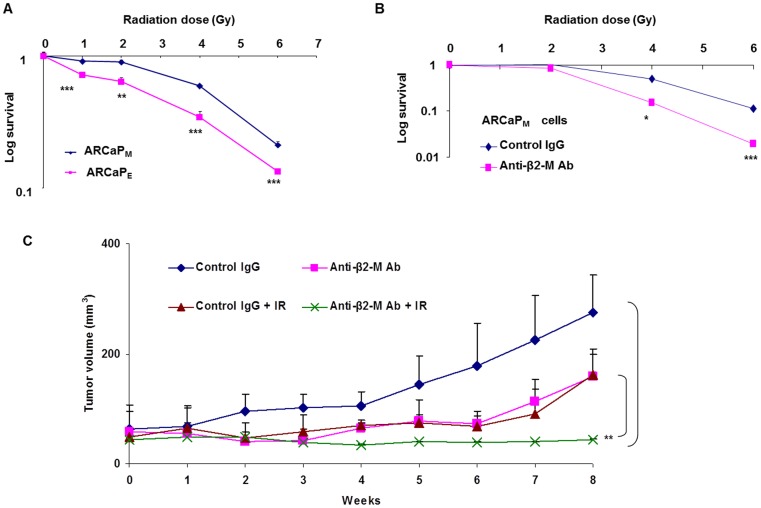
Figure 1. Anti-β2-M Ab sensitizes prostate cancer cells to radiation *in vivo*. **A.** Radiation sensitivity of ARCaP_E_ and ARCaP_M_ prostate cancer cells by clongenic assay. (** p<0.01, *** p<0.001, Student’s t test). **B.** ARCaP_M_ cells are sensitized to radiation in the presence of anti-β2-M Ab using clongenic assay. (*p<0.03, *** p<0.008, ANOVA). **C.** Effect of anti-β2-M Ab (0.8 mg/kg) and radiation (15 Gy) on tumor growth in subcutaneous ARCaP_M_ xenograft nude mice model. (** p<0.01, Student’s t test).

**Figure 2:**
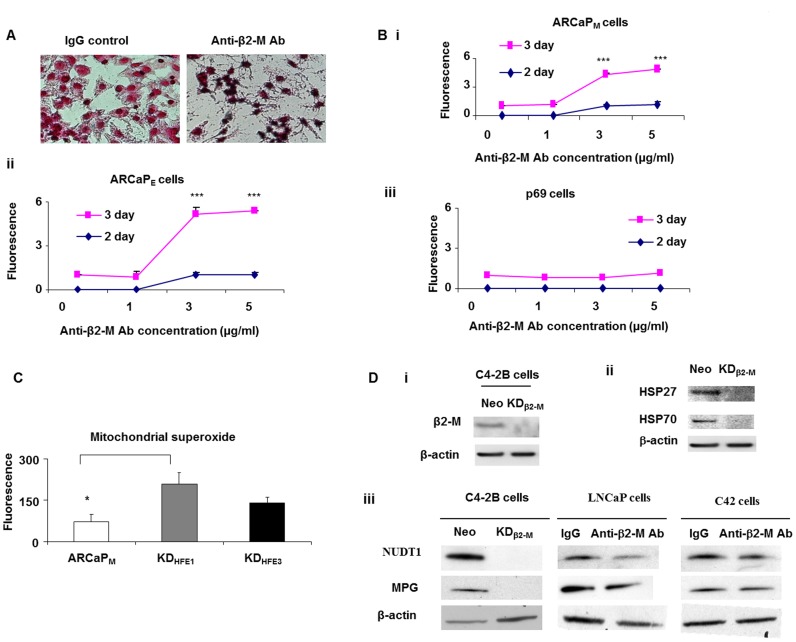
Anti-β2-M Ab increases iron and decreases DNA repair enzymes in prostate cancer cells. **A.** Iron staining in control and anti-β2-M Ab (5 µg/ml) treated cells using iron staining kit in ARCaP_M_ prostate cancer cell lines. **B.** Mitochondrial superoxide levels in response to anti-β2-M Ab treatment in a time and dose dependent manner in **i.** ARCaP_M_, **ii.** ARCaP_E_ prostate cancer cell lines (***p<0.001, Student’s t test) and **iii.** p69 immortalized prostate epithelial cells using MitoSOX dye. **C.** Mitochondrial superoxide in ARCaP_M_, KD_HFE1_ and KD_HFE3_ using MitoSOX dye (*p<0.05, Student’s t test). **D.** Expression of stress response proteins and DNA repair enzymes in C4-2B Neo control and β2-M knockdown cell lines. **i.**β2-M protein expression, **ii.** HSP27 and HSP70 protein expression and **iii.** NUDT1 and MPG protein expression. NUDT1 and MPG protein expression in response to anti- β2-M Ab treatment in LNCaP and C4-2 prostate cancer cells.

**Figure 3:**
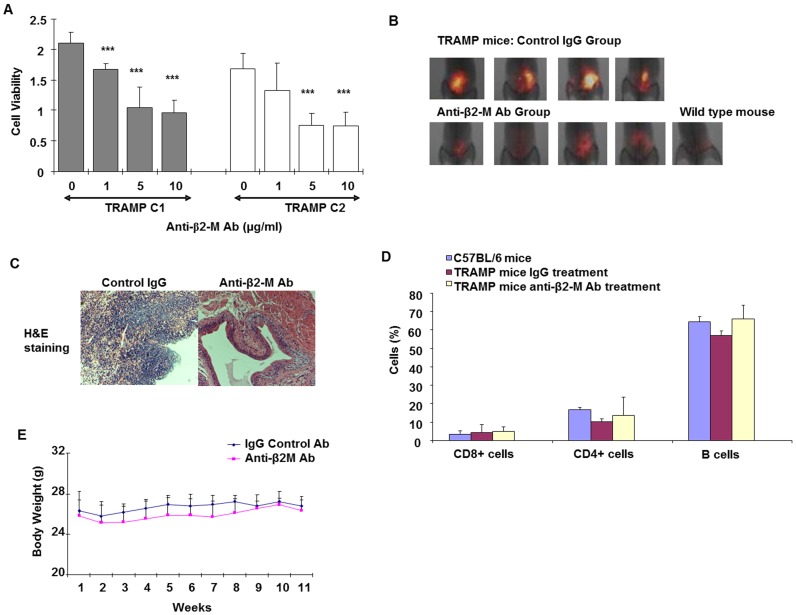
Anti-β2-M Ab prevents tumor formation in spontaneous prostate cancer TRAMP mouse model. **A.** Cell viability of TRAMP C1 and TRAMP C2 prostate cancer cells in response to anti-β2-M Ab. (***p<0.001, Student’s t test). **B.** Merged near infra-red and X-ray image of abdomen of TRAMP mice treated with control IgG and anti-β2-M Ab (n = 4). Representative parental mice used as additional control (C57BL/6 mice). The tumorigenecity of control IgG antibody group was 100% (n = 4) and the tumorigenecity of anti-β2-M Ab treated group was 25% (n = 4). **C.** H&E images of prostates of control IgG mice and anti-β2-M Ab treated mice (10X). **D.** Immune cell (T and B cells) numbers of wild type mice, control IgG mice and anti-β2-M Ab treated mice measured by flow cytometry. **E.** Body weights of TRAMP mice treated with IgG or anti-β2-M Ab.

**Figure 4:**
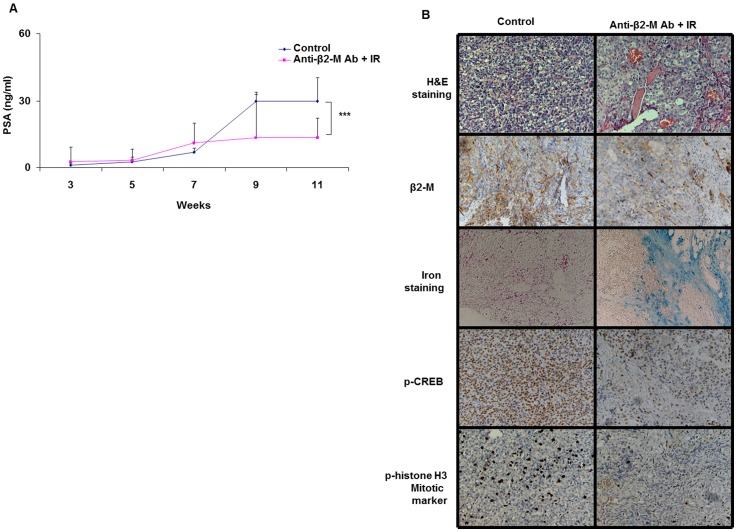
Combination treatment of anti-β2-M Ab and radiation in intra-tibial mouse model of prostate cancer. Tumor incidence in mice tibias were analyzed from H&E images and x-ray scans. The tumorigenecity of control group was 94% (n = 18 tibias) and the tumorigenecity of anti-β2-M Ab+irradiation (IR) treated group was 67% (n = 18 tibias). **A.** Tumor progression in control and combination treatment (anti-β2-M Ab and irradiation) analyzed by PSA measurements (ng/ml). (***p<0.006, Student’s t test). **B.** Immunohistochemical staining of tibias in control and combination treatment group stained for H&E, β2-M protein, iron staining, p-CREB and p-histone H3 (10X).

**Figure 5:**
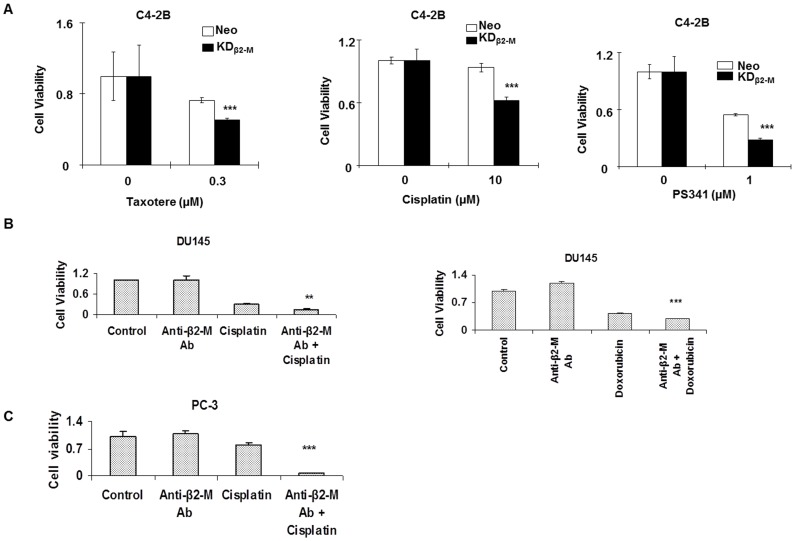
Anti-β2-M Ab sensitizes prostate cancer cells to chemotherapeutic cancer drugs. **A.** Cell viability of C4-2B Neo control cells and KD_β2-M_ cells in response to: taxotere (0.3 µM), cisplatin (10 µM) and PS341 (1 µM). (***p<0.001, Student’s t test) **B.** Cell viability of DU154 prostate cancer cells in response to combination of anti-β2-M Ab (0.5 µg/ml) and cisplatin (100 µM)/doxorubicin (100 µM). (***p<0.001, Student’s t test). **C.** Cell viability of PC-3 prostate cancer cells in response to combination of anti-β2-M Ab (1 µg/ml) and cisplatin (100 µM). (***p<0.001, Student’s t test).

The antibody used in Figures 1, 2 and 5 is from Santa Cruz Biotechnology. Since the antibody solution had 0.005% final concentration of sodium azide and gelatin, we tested if sodium azide or gelatin was toxic to these cells. ARCaP_M_ prostate cancer cells were not affected by high doses (0.1%) of sodium azide or gelatin (Figure S1). The antibody used in Figure 3 and 4 is from mice ascites produced from BBM.1 hybridoma (ATCC). The IgG antibody was purified using a Melon gel IgG purification Kit (Fisher Scientific) and antibody levels were quantified using nanodrop (Thermo Scientific). Iron staining of cells treated with IgG and anti-β2-M Ab was performed with an iron staining kit (Sigma). LNCaP and C4-2 cancer cells were used to detect DNA repair proteins in response to anti-β2-M Ab. Cells were treated with anti-β2-M Ab (10 µg/ml) for 24 h. Mouse TRAMP (C1 and C2) prostate cancer cells were tested with increasing concentrations of anti-β2-M Ab (0–10 µg/ml) and their cell viability was examined.

### In Vivo Animal Experiments

#### Subcutaneous Xenograft Study

Four-week-old male nude mice ((NCRNU, Taconic) were subcutaneously injected with 2×10^6^ ARCAP_M_ prostate cancer cells with matrigel (1∶1) in the flank. When the tumor reached 4 mm^3^, mice were treated with IgG or anti-β2-M Ab in gelform®. The gelform® was immersed in antibody (0.8 mg/kg) and surgically implanted adjacent to the tumors. Twenty four hours later tumors were irradiated with 15 Gy. Each group had five mice. Tumor volume was measured weekly.

#### Tramp Mice Study

TRAMP mice were obtained from the Emory core mouse facility. Parental C57BL/6 mice were also used as controls. Previous studies demonstrated anti-β2-M Ab was toxic to TRAMP prostate cancer cells *in vitro*. Mice 21–26 weeks of age were paired age-wise and separated into a control IgG and anti-β2-M Ab group. Starting from 21/26 weeks to 32/37 weeks mice were given IgG Ab or anti-β2-M Ab (8 mg/kg) every three days for a total of 11 weeks. Body weights were measured weekly. Tumor development was monitored using the near-infrared dye (NIR) IR-783 15 using a Kodak imaging station 4000 MM machine. X-ray images were taken simultaneously and superimposed to determine tumor location. Mice were sacrificed at 33/38 weeks and prostates were removed, fixed, and sectioned, and H&E was performed 4.

#### Intra-Tibial Study

Four-week-old male nude mice (NCRNU) (Taconic) were injected with C4-2 prostate cancer cells (1×10^6^ cells) suspended in 10 µl sterile PBS into both tibias (n = 18). One week after injection, anti-β2-M Ab (8 mg/kg) was injected intra-peritonially once every 3 days for the rest of the study. Tumor progression was determined bi-weekly, using prostate specific antigen (PSA) marker detection. Serum PSA was measured by microplate ELISA using an Abbott IMx machine (Abbott Park, IL). Nine weeks after tumor injection, the tibias were irradiated with 4 Gy on three consecutive days, receiving a total of 12 Gy. Anti-β2-M Ab treatment was given prior to the irradiation treatment on all three days. Anti-β2-M Ab treatment was continued every 3 days after the irradiation treatment until week 11. A schematic of the treatment protocol is included in Figure S2. On week 12 the mice were sacrificed and the tibias were sent for pathology. Tibias were harvested and H&E and iron staining (Iron stain kit, Sigma) was performed. Immunohistochemical staining for β2-M (Santa Cruz Biotechnology), p-CREB (Cell Signaling Technology), and p-histone H3 (Millipore) were performed as previously described 2.

Immune cell study: Splenocytes were prepared by crushing spleens. Cells were washed and incubated with specific antibodies as previously described 16. The antibodies used were PE-Cy5 anti-CD45R/B220 (RA3-6B2), CD3-APC, CD4-PE and CD8-FITC obtained from eBiosciences. Analyses were conducted on a dual laser flow cytometer (FACSCalibur) 16.

### Reactive Oxygen Species Studies

Mitochondrial superoxide was detected using MitoSOX (Molecular Probes, Eugene, OR). Samples were incubated for a minimum of 40 min at 37°C in the dark on a rotator and fluorescence was measured.

### Stable Knockdown Of β2-M And Hfe In Arcap_M_ Cells

Control and β2-M siRNA was retrovirally transfected into ARCaP_M_ cells. Β2-M knockdown cells are indicated as KDI and KDII. Lentiviral transduction was performed to inhibit HFE, as per instructions (Sigma, St. Louis, MO). Cells were selected using puromycin (4 µg/ml) as previously reported 4. Negative control cells which did not receive the viral particles died in 3–5 days. HFE shRNA transduced cells were characterized for HFE levels 7–10 days after transduction. C4-2B (Neo) control and C4-2B β2-M knockdown cells (KD _β2-M_) were generated previously 2.

### Statistical Analysis

All experiments were performed in triplicate at least two independent times. Values were expressed as means ± standard deviation. Statistical analysis was performed using Student’s *t*-test or ANOVA. Values of p<0.05 were considered to be statistically significant.

## Results

### Anti-β2-M Ab Sensitizes Prostate Cancer Cells To Radiation *In Vivo*

Previous studies demonstrate that β2-M and HFE play an important role in cancer progression 4. Inhibition of β2-M, using anti-β2-M Ab has been shown to induce cell death in several cancers including prostate cancer. More than 50% of cancer patients invariably undergo radiation therapy during the course of disease progression. However, radiation treatment has adverse effects. Targeted therapies including therapeutic antibodies could potentially act as radiosensitizing agents. To test the hypothesis that treatment with anti-β2-M Ab will sensitize prostate cancer cells to radiation, we used the well characterized ARCaP prostate cancer model which metastasizes to the bone in mouse xenograft models. ARCaP_E_ cell line is epithelial-like and has low propensity for metastasis and also expresses low levels of β2-M and the ARCaP_M_ cell line, is mesenchymal-like and by contrast, is highly metastatic to bone and expresses high levels of β2-M 4. The radiation sensitivity was determined using clonogenic assay. We demonstrate that ARCaP_M_ cells are more resistant to radiation compared to ARCaP_E_ cells (Figure 1A). To determine if β2-M is involved in radiation resistance, we generated β2-M knockdown stable ARCaP_M_ prostate cancer cells (clones KDII and KDI). We performed a clonogenic assay to determine their radiation sensitivity. Both KDI and KDII were more sensitive to radiation treatment compared to ARCaP_M_ control cells (Figure S3A). In addition to the genetic approach, we used anti-β2-M Ab to inhibit β2-M prior to radiation therapy. The combination treatment of anti-β2-M Ab (3 µg/ml) and radiation had a synergistic effect on prostate cancer cell death in vitro (Figure 1B). Synergism was analyzed by ANOVA, and anti-β2-M Ab and radiation had a synergistic effect at 4 Gy and 6 Gy doses of radiation. Since β2-M interacts with HFE to mediate its cellular processes 4, we knocked down HFE in ARCaP_M_ prostate cancer cells using lentiviral shRNA particles. HFE expression was decreased in HFE knockdown cells (clones KD_HFE1_ and KD_HFE3_) compared to control ARCaP_M_ cells (Figure S3B). Inhibition of HFE also decreased β2-M expression, and thus β2-M/HFE complexes. The radiation response of KD_HFE1_ and KD_HFE3_ cells was determined using a clonogenic assay. KD_HFE1_ and KD_HFE3_ cells were more sensitive to radiation compared to control ARCaP_M_ prostate cancer cells.

To determine if anti-β2-M Ab and irradiation synergize *in vivo*, we injected ARCaP_M_ cells sub-cutaneously into the flanks of nude mice. Once tumors reached a size of 4 mm^3^ the xenografts were surgically implanted with anti-β2-M Ab IgG (0.8 mg/kg) in gelform®. Twenty-four hours later tumors were irradiated with 15 Gy. Each group had five tumors and the tumor volume was measured weekly. Anti-β2-M Ab and radiation alone partially decreased tumor growth. However, in the combination treatment group, none of the tumors grew in the mice (Figure 1C). These results demonstrate that anti-β2-M Ab is an effective agent for prostate cancer treatment, and combination treatment with anti-β2-M Ab and radiation is significantly more effective than antibody only or radiation only treatment.

### Inhibition Of β2-M Increases Iron Overload, Reactive Oxygen Species And Decreases Dna Repair Enzymes And Stress Response Proteins

Transgenic mice lacking β2-M or HFE have increased iron overload 11. β2-M/HFE form a complex and interact with transferrin receptor (TFRC1) 8,9. The β2-M/HFE complex inhibits the formation of transferrin-TFRC1 complexes. Thus, iron which is bound to transferrin is prevented from entering the cell and therefore mice lacking β2-M of HFE have increased iron overload. We tested if anti-β2-M Ab could induce iron overload and reactive oxygen species (ROS) in prostate cancer cells. ARCaP_M_ cells were treated with anti-β2-M Ab (5 µg/ml for 24 h) and iron content was determined using Prussian blue iron staining. Increased dark blue-black staining of iron was observed in anti-β2-M Ab treated cells compared to isotype control treated ARCaP_M_ prostate cancer cells (Figure 2A). To determine if anti-β2-M Ab induced increased reactive oxygen species (ROS) as a result of increase in iron overload, we tested for levels of mitochondrial superoxide using MitoSOX. Two prostate cancer cells (ARCaP_M_ and ARCaP_E_) and p69 immortalized normal prostate epithelial cells were used to test this hypothesis. An increase in mitochondrial superoxide, a reactive oxygen species, was observed in the prostate cancer cells and not in the normal cells in a dose and time dependent manner in response to the anti-β2-M Ab (Figure 2B). Previous studies demonstrate that HFE knockdown cells have increased basal iron 4. We tested if inhibition of HFE in prostate cancer cells would alter mitochondrial superoxide levels. The basal level of mitochondrial superoxide was measured using MitoSOX and we found that the basal levels were increased in HFE knockdown clones (KD_HFE1_ and KD_HFE3_) compared to the control (Figure 2C). Radiation resistance is increased by elevated DNA repair enzymes and stress response proteins. Next, we sought to determine changes in stress response proteins in β2-M knockdown prostate cancer cells. Using C4-2B control and β2-M knockdown prostate cancer cells (KD_β2-M_) we tested the levels of stress response proteins such as heat shock protein 27 (HSP27) 17 and heat shock protein 70 (HSP70) 18 and DNA repair enzymes such as N-methylpurine-DNA glycosylase (MPG) 19 and nudix (nucleoside diphosphate linked moiety X)-type motif 1 (NUDT1) 20. Interestingly, the stress response and heat shock proteins were downregulated in β2-M knockdown clones KD_β2-M_ (Figure 2D). Additionally, prostate cancer cells were treated with anti-β2-M Ab (10 µg/ml) for 24 h and the protein levels of DNA repair enzymes MPG and NUDT1 were examined. Anti-β2-M Ab moderately decreased the levels of MPG and NUDT1 proteins. These studies demonstrate that anti-β2-M Ab induces several cytotoxic effects such as iron overload, increased free radical levels, decreased DNA repair enzymes and stress response proteins in prostate cancer cells and thereby sensitize prostate cancer cells to radiation.

### Anti-β2-M Ab Prevents Tumor Growth In A Spontaneous Immuno-Competent Transgenic Adenocarcinoma Of Mouse Prostate (tramp) Mice Model

TRAMP C1 and TRAMP C2 prostate cancer cells 21 are cell lines derived from spontaneous mouse model of adenocarcinoma. We performed in vitro studies to test the effect of anti-β2-M Ab in the TRAMP cell lines. Both TRAMP C1 and TRAMP C2 murine prostate cancer cells undergo cell death with increasing concentrations of anti-β2-M Ab (Figure 3A). Next, we tested the effects of the antibody *in vivo*. TRAMP mice (age 21 to 26 weeks) were paired and treated either with a control IgG or anti-β2-M Ab group (n = 4). Parental mice (C57BL/6 mice) were maintained until the end of the experiment. Starting at 21/26 weeks, mice were given 8 mg/kg of IgG Ab or anti-β2-M Ab every three days until the mice reached 32/37 weeks and were sacrificed a week later. Body weights of mice were determined weekly. Tumor development was monitored using near infrared dye (IR-783) 15 biweekly. Imaging was performed using infra-red imaging and X-ray imaging with a Kodak imaging machine. After the mice were euthanized the prostates were dissected and stained using H&E. We found that three out of four mice in the control IgG group developed tumors and one had hyperplasia, as confirmed by H&E and infrared imaging (Figure 3B, 3C). Interestingly, three out of four mice had no tumor and one mice developed hyperplasia in the anti-β2-M Ab treated group, as confirmed by H&E and infra-red imaging (Figure 3B, 3C). Thus the tumorigenecity of the control group was 100% and of the anti-β2-M Ab was 25%. Since β2-M is expressed by cells of the immune system, we measured the possible immunotoxicity of continuous treatment with anti-β2-M Ab. We demonstrate that treatment with anti-β2-M Ab did not affect immune cell numbers (CD8+ and CD4+ T cells and B cells) and body weight of mice. T and B cell numbers were not affected by anti-β2-M Ab treatment compared to IgG or parental mice (Figure 3D). The body weights were also not affected when anti-β2-M Ab was given continuously every three days for 11 weeks (Figure 3E). These studies demonstrate that anti-β2-M Ab treatment does not compromise the immune system and the body weight of the mice and that it prevents tumor development in spontaneous prostate mouse models of prostate cancer.

### Combination Treatment With Anti-β2-M Ab And Irradiation Reduces Prostate Cancer Growth In The Bone Microenvironment

The second most prevalent site for prostate cancer bone metastasis is the bone. Currently there are no good treatments for prostate cancer growth in the bone. Therefore, we tested the efficacy of anti-β2-M Ab and irradiation on prostate cancer growth in the bone. To test this, we injected androgen independent C4-2 prostate cancer cells intra-tibially into nude mice. One week after tumor inoculation in the bone, mice were administered anti-β2-M Ab (8 mg/kg) (n = 9 mice) intra-peritoneally every three days for 11 weeks or phosphate buffered saline (n = 9 mice). Prostate specific antigen (PSA) levels in the serum of mice and the body weight of the mice were measured biweekly. At 9 weeks, the anti-β2-M Ab treatment group was given 4 Gy irradiation for three consecutive days (12 Gy in total) (Figure S2). Prior to radiation, mice were given a dose of anti-β2-M Ab (8 mg/kg). The mice were maintained until 12 weeks after tumor injection and sacrificed. The presence of tumor cells was determined by H&E staining. Anti-β2-M Ab prevented tumor formation in 33% of the tibias inoculated with the tumor cells. The control mice had 94% tumor incidence and the anti-β2-M Ab plus irradiation treated group had 67% tumor incidence. Treatment with the anti-β2-M Ab also delayed tumor development, which was evident by a decrease in PSA levels in these mice. The majority (7/9) of the control mice had detectable PSA at 3 weeks after intra-tibial injection, whereas the anti-β2-M Ab treated group had delayed tumor formation and less detectable PSA levels (3/9 at 3 weeks after tumor injection). At 9 weeks after radiation, there was a significant decrease in the PSA level of antibody treated mice compared to the control mice (p<0.006) (Figure 4A). Using immunohistochemistry we demonstrate decreased β2-M staining in the anti-β2-M Ab+irradiation treated group compared to the control group (Figure 4B). Moreover, the anti-β2-M Ab and irradiation treated group had significantly increased iron staining in the bone (42%) compared to control mice (6%) (Figure 4B), suggesting iron overload in antibody treated group. We also looked at the downstream pathways targeted by the anti-β2-M Ab and found that there is a decrease in the levels of these targets (p-CREB) in the tibia of the antibody and radiation treated mice compared to the control 2. Additionally, anti-β2-M Ab and radiation treated group had decreased mitosis, indicated by the mitotic marker, p-histone H3 (Figure 4B). Prolonged treatment with anti-β2-M Ab was not toxic to the mice as the body weight of the mice was stable (Figure S4). Taken together, these studies demonstrate that an anti-β2-M Ab and irradiation combination treatment can reduce tumorigenecity and significantly delay and/or inhibit growth of prostate cancer cells in the bone.

### Inhibition Of β2-M Sensitizes Prostate Cancer Cells To Chemotherapeutic Agents

Since inhibition of β2-M results in iron overload, increase in reactive oxygen species and decreases in stress response proteins *in vitro*, we tested if treatment with anti-β2-M Ab could sensitize prostate cancer cells to clinically used chemotherapeutic agents. Using C4-2B and C4-2B β2-M knockdown prostate cancer cells (KD_β2-M_) 2, we tested if the β2-M knockdown cells were more sensitive to taxotere, cisplatin and PS341. β2-M expression was low in KD_β2-M_ cells compared to Neo (control) cells (Figure 2D). Inhibition of β2-M significantly sensitized prostate cancer cells to taxotere (0.3 µM), cisplatin (10 µM) and PS341 (1 µM) (Figure 5A). Anti-β2-M Ab sensitized DU145 cells to cisplatin and doxorubicin (Figure 5B) and PC-3 cells to cisplatin (Figure 5C). Using bliss independence analysis a synergistic interaction was observed in DU145 cells treated with anti-β2-M Ab and doxorubicin and in PC-3 cells treated with anti-β2-M Ab and cisplatin.

These studies demonstrate that anti-β2-M Ab is a promising agent for combination therapy with commonly used treatments in cancer such as radiation and chemotherapy. Since prostate cancer bone metastasis is difficult to treat, combination treatments with anti-β2-M Ab maybe more effective in reducing tumor burden.

## Discussion

Prostate cancer is the second leading cause of death among men in North America. Elevated β2-M expression is associated with the progression of human prostate cancer 22, breast cancer 23, renal cancer 24, lung cancer 25, colon cancer 26 and a number of liquid tumors such as multiple myeloma, lymphoma and leukemia 3. β2-M mediates epithelial to mesenchymal transition, and cancer metastasis to bone and other soft tissues 4. Therefore elevated β2-M tissue levels indicates poor prognosis. Thus, it is important to target β2-M in prostate cancer patients to prevent metastasis. Previously, we and others demonstrated that treatment with anti-β2-M Ab induced cancer cell death in both solid and liquid tumors 3,6,27. Since inhibition of β2-M leads to decreased stress response, we hypothesized that a combination treatment of anti-β2-M Ab with radiation or chemotherapy can enhance the cancer cell kill (radiosensitization and chemosensitization). Inhibition of either β2-M or HFE in prostate cancer cells leads to their radiosensitization (Figure 1B, Figure S3A, S3B). Using spontaneous prostate cancer TRAMP tumor model, we also demonstrate that anti-β2-M Ab alone, prevents or delays tumor growth with no toxic side effects (Figure 3). Using a subcutaneous xenograft mouse model and an intra-tibial bone mouse model we demonstrate that the combination treatment of anti-β2-M Ab and radiation is more effective for treating tumor compared to antibody or radiation only treatment approach (Figure 1C, Figure 4). Thus, we demonstrate that anti-β2-M Ab in combination with irradiation significantly inhibits tumor growth *in vitro* and *in vivo* and in immune-deficient and in immune-competent mice. Current treatments do not specifically target the cancer cells in the bone microenvironment. Therefore, we propose that anti-β2-M Ab is a promising agent in aggressive prostate cancer bone metastatic patients and therefore combination treatment with the antibody and radiation will reduce tumor burden in such patients.

β2-M has been previously shown to activate several pathways in cancer cells such as protein kinase A 28, vascular endothelial growth factor 29, androgen receptor 7, fatty acid synthase 7 and lipid raft signaling pathways 3. In this study we demonstrate that β2-M regulates the cellular balance of iron and reactive oxygen species (Figure 2, 4B). Additionally, β2-M also regulates the expression of stress response proteins such as HSP27 and HSP70 and DNA repair enzymes NUDT1 and MPG (Figure 2). Thus, decreased stress response proteins make the cancer cells susceptible to cellular damage. Additionally in the absence of β2-M resulted in the absence of several DNA repair enzymes, possibly resulting in increased DNA damage. Thus, β2-M inhibited cells are very sensitive to treatments such as radiation and chemotherapy, since they lack the ability to respond to cellular damage.

Several patients suffer from hemochromatosis, due to mutations in iron homeostasis pathways. Most hemochromatosis patients have a mutation in HFE at C282Y, which is a binding site between HFE and β2-M. In these patients β2-M/HFE complexes are not formed, and this leads to multi-organ iron overload diseases. Consistent with our findings, hemochromatosis (HH) patients are sensitive to radiation 30. Previous studies demonstrate that 1∶8 Caucasians have HFE heterozygous mutations. However, heterozygous β2-M knockout mice do not produce iron overload conditions like homozygous β2-M knockout mice 10. Iron overload caused cancer (hepatocellular) in some organs and regression in some, such as the prostate 10. HH patients who have iron overload have also been shown to develop hypogonadism 10. These observations suggest that iron overload results in regression of the prostate gland 10. Additionally, prostate cancer patients have low iron load compared to the population in general 31.

### Conclusions

β2-M is highly expressed in tissues of prostate cancer bone metastasis patients. Overexpression of β2-M and has been shown to induce bone metastasis in prostate, breast, renal and lung cancer. In this study we target β2-M using anti-β2-M Ab and in combination with radiation or chemotherapy using bone xenograft mouse models. A combination of anti-β2-M Ab sensitizes prostate cancer cells to radiation and chemotherapy. Anti-β2-M Ab induces increased iron and reactive oxygen species and decreases stress response proteins and DNA repair enzymes in prostate cancer cells. Thus, anti-β2-M Ab can sensitize cancer cells to radiation. Therefore, anti-β2-M Ab is a promising agent which can be used with radiation or chemotherapy for patients suffering from prostate cancer bone metastasis.

## Supporting Information

Figure S1 **Cell survival of ARCaP_M_ cells in response to gelatin and sodium azide using MTS assay.**
(TIF)

Figure S2 *In vivo* experiment timeline. Mice were injected with C4-2 prostate cancer cells intra-tibially. One week later mice were given anti-β2-M Ab (8 mg/kg) intra-peritonially every third day for 11 weeks. At ninth week mice were given a dose of anti-β2-M Ab (8 mg/kg) and then irradiated with 4 Gy on three consecutive days. Mice were sacrificed at week 12.
(TIF)

Figure S3 **A.** Radiation sensitivity in β2-M knockdown cells (KDI and KDII) compared to controls using clongenic assay. β2-M expression levels in these cell lines. **B.** Clongenic survival of ARCAP_M_, HFE knockdown prostate cancer cells (KD_HFE1_ and KD_HFE3_). Western analysis of HFE and β2-M in HFE knockdown prostate cancer cells (KD_HFE1_ and KD_HFE3_).
(TIF)

Figure S4 **Body weights in grams during the course of the** ***in vivo*** **intra-tibial experiment.**
(TIF)
