# Supplementary figures and images for: Inhibition of β2-Microglobulin/Hemochromatosis Enhances Radiation Sensitivity by Induction of Iron Overload in Prostate Cancer Cells
Source: PLoS One. 2013 Jul 10;8(7):e68366. doi: 10.1371/journal.pone.0068366 (PMC3707913; doi:10.1371/journal.pone.0068366)

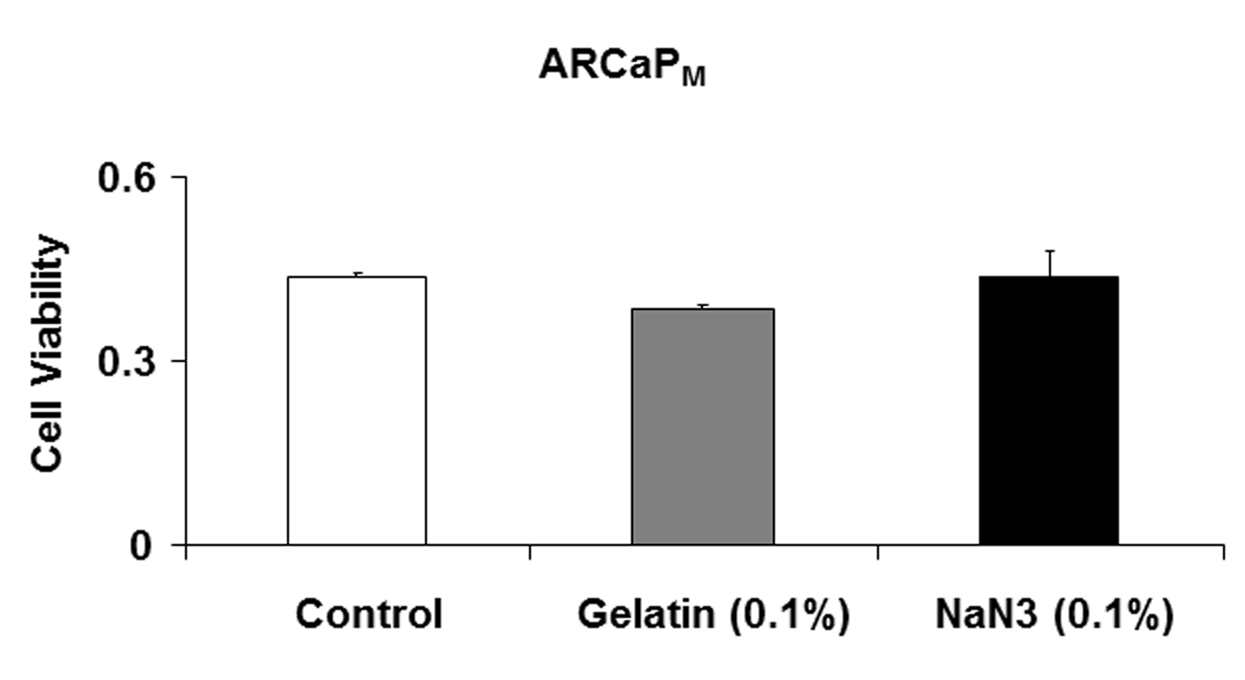

Supplement: Figure S1 — (TIF) [file pone.0068366.s001.tif]

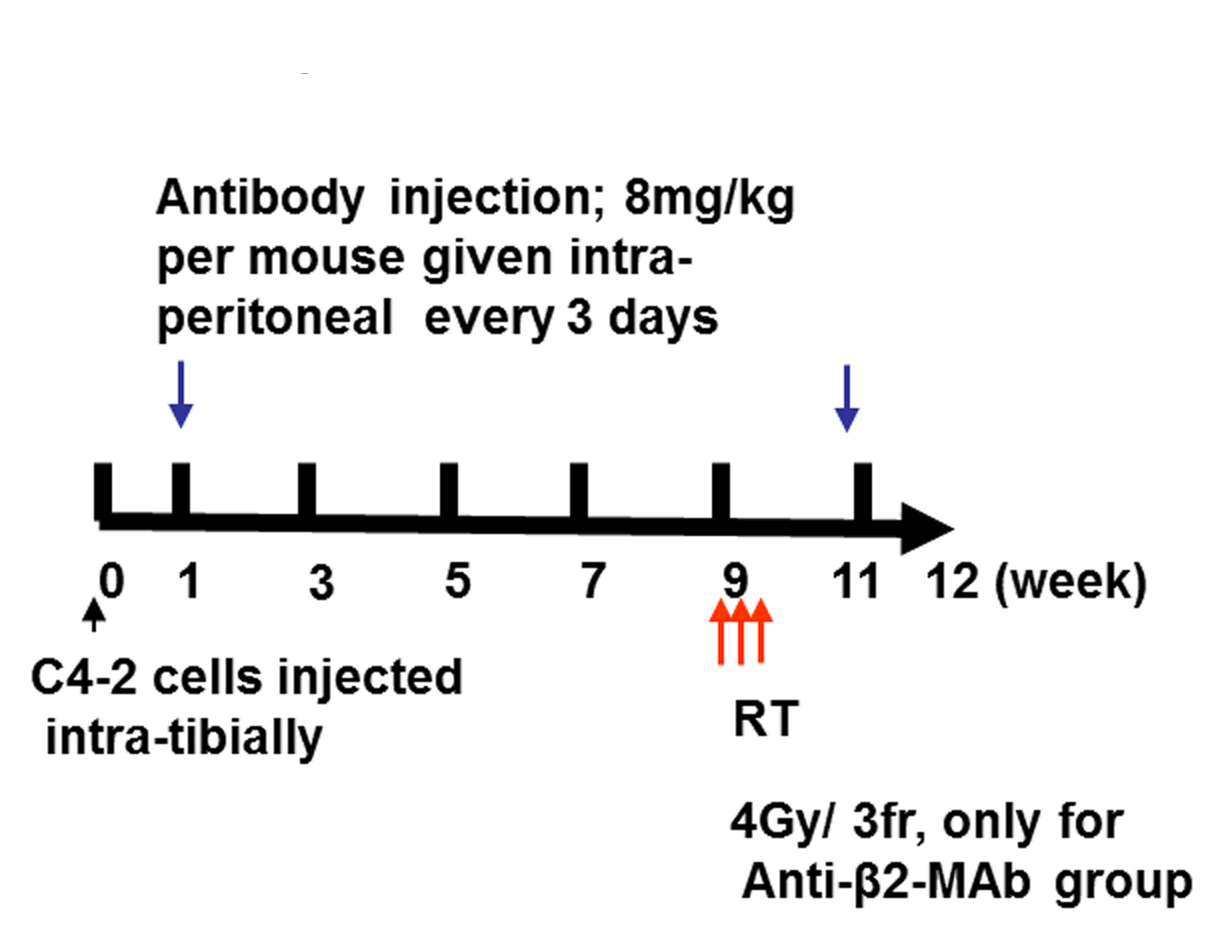

Supplement: Figure S2 — Mice were injected with C4-2 prostate cancer cells intra-tibially. One week later mice were given anti-β2-M Ab (8 mg/kg) intra-peritonially every third day for 11 weeks. At ninth week mice were given a dose of anti-β2-M Ab (8 mg/kg) and then irradiated with 4 Gy on three consecutive days. Mice were sacrificed at week 12. (TIF) [file pone.0068366.s002.tif]

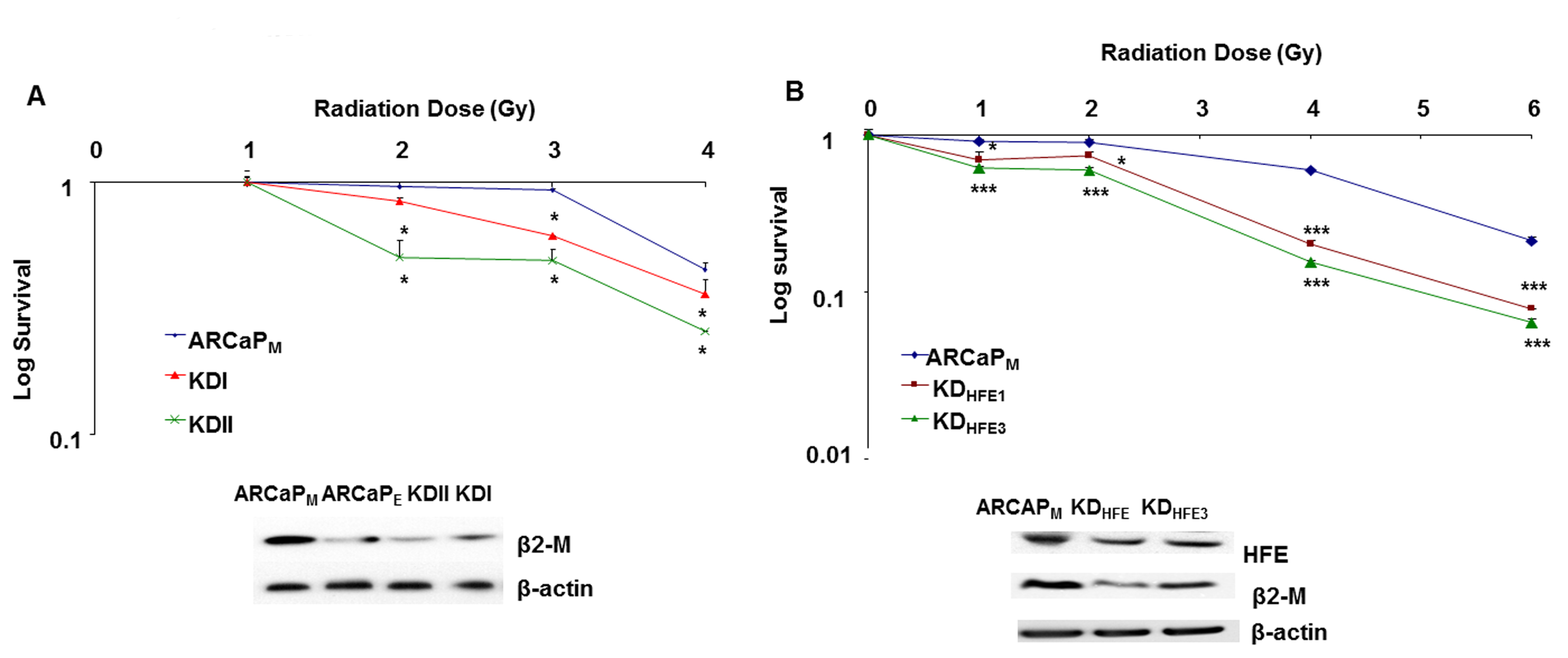

Supplement: Figure S3 — A. Radiation sensitivity in β2-M knockdown cells (KDI and KDII) compared to controls using clongenic assay. β2-M expression levels in these cell lines. B. Clongenic survival of ARCAPM, HFE knockdown prostate cancer cells (KDHFE1 and KDHFE3). Western analysis of HFE and β2-M in HFE knockdown prostate cancer cells (KDHFE1 and KDHFE3). (TIF) [file pone.0068366.s003.tif]

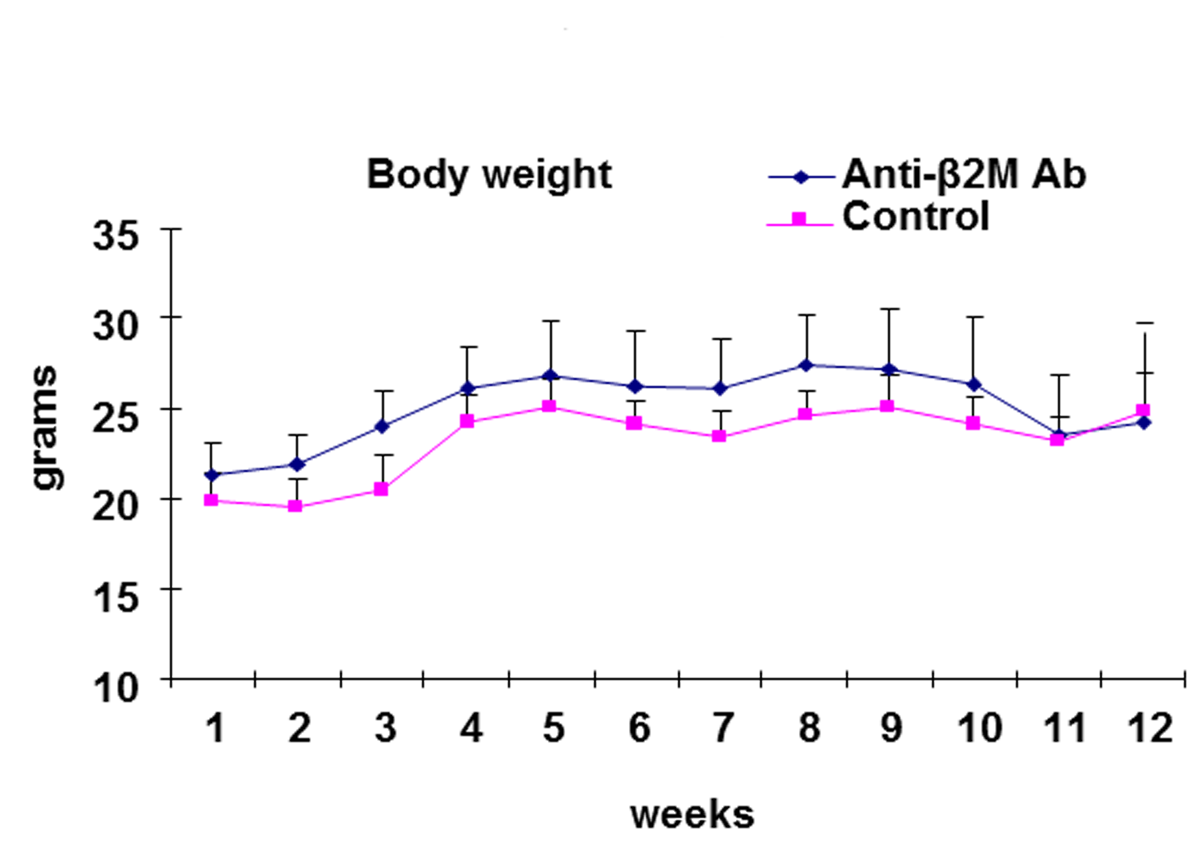

Supplement: Figure S4 — (TIF) [file pone.0068366.s004.tif]
